# Supplementary material for: Associations between Measured and Patient-Reported Physical Function and Survival in Advanced NSCLC
Source: Healthcare (Basel). 2022 May 17;10(5):922. doi: 10.3390/healthcare10050922 (PMC9141394; doi:10.3390/healthcare10050922)
Supplement: Supplementary file 1 [file healthcare-10-00922-s001.zip › healthcare-1698452-supplementary.pdf]

Table S1 Physical performance as predictor of disease control

				Univariable analysis			Multivariable model with TUG			Multivariable model with 5mWT			Multivariable model with PRPF		
		**n**	**(%)**	**OR**	**95% CI**	**p**	**OR**	**95% CI**	**P**	**OR**	**95% CI**	**p**	**OR**	**95% CI**	**p**
**TUG***		179	(100%)	0.98	0.90-1.07	0.66	0.95	0.87-1.05	0.30						
**5mWT***		179	(100%)	0.94	0.83-1.07	0.36				0.98	0.89-1.08	0.69			
**PRPF***		149	(83%)	1.00	0.98-1.02	0.94							1.02	1.00-1.03	0.13
**Age***		179	(100%)	1.01	0.97-1.05	0.69	1.02	0.98-1.07	0.36	1.02	0.98-1.06	0.39	1.02	0.97-1.07	0.42
**Sex**	**Male**	86	(48%)	1			1			1			1		
	**Female**	93	(52%)	0.59	0.32-1.08	0.09	0.61	0.32-1.13	0.12	0.61	0.33-1.14	0.12	0.76	0.39-1.47	0.41
**Stage of disease**	**IIIB**	11	(6%)	1			1			1			1		
	**IV**	168	(94%)	0.59	0.32-1.08	0.09	0.84	0.22-3.18	0.12	0.82	0.22-3.07	0.77	0.55	0.14-2.20	0.40
**WHO PS**	**0**	58	(32%)	1			1			1			1		
	**1**	103	(58%)	0.42	0.21-0.84	0.02	0.42	0.20-0.86	0.02	0.41	0.20-0.84	0.01	0.61	0.29-1.32	0.21
	**2**	18	(10%)	0.44	0.15-1.31	0.14	0.54	0.15-2.00	0.36	0.42	0.13-1.42	0.16	0.61	0.18-2.07	0.43

*CR, PR and SD was defined as disease control. *Entered as continuous variables*
